# Highly efficient production of 2,3-butanediol from xylose and glucose by newly isolated thermotolerant *Cronobacter sakazakii*

**DOI:** 10.1186/s12866-022-02577-z

**Published:** 2022-06-24

**Authors:** Chansom Keo-oudone, Koudkeo Phommachan, Orathai Suliya, Mochamad Nurcholis, Somchanh Bounphanmy, Tomoyuki Kosaka, Mamoru Yamada

**Affiliations:** 1 Department of Biology, Faculty of Natural Science, National University of Laos, Lao PDR, 7322 Vientiane, Laos; 2 Graduate School of Sciences and Technology for Innovation, Yamaguchi University, 753-8515 Yamaguchi, Japan; 3 Department of Food Science and Technology, Faculty of Agricultural Technology, Brawijaya University, 65145 Malang, Indonesia; 4 Department of Biological Chemistry, Faculty of Agriculture, Yamaguchi University, 753-8515 Yamaguchi, Japan; 5 Research Center for Thermotolerant Microbial Resources, Yamaguchi University, 753-8515 Yamaguchi, Japan

**Keywords:** 2,3-Butanediol, *Cronobacter sakazakii*, Xylose-fermenting microbe, High-temperature fermentation, Lignocellulosic biomass

## Abstract

**Background:**

2,3-Butanediol (2,3-BD), a valuable compound used for chemicals, cosmetics, pesticides and pharmaceuticals, has been produced by various microbes. However, no high-temperature fermentation of the compound at high productivity has been reported.

**Methods:**

Thermotolerant xylose-utilizing microbes were isolated from 6 different districts in Laos and screened for a low accumulation of xylitol in a xylose medium at 37 ˚C. One isolate was found to produce 2,3-BD and identified by 16S rDNA sequencing. The 2,3-BD fermentation capacity was investigated at different temperatures using xylose and glucose as carbon sources, and the fermentation parameters were determined by a high-performance liquid chromatography system.

**Results:**

By screening for a low accumulation of xylitol in a xylose medium, one isolate that accumulated almost no xylitol was obtained. Further analyses revealed that the isolate is *Cronobacter sakazakii* and that it has the ability to produce 2,3-BD at high temperatures. When xylose and glucose were used, this strain, named *C. sakazakii* OX-25, accumulated 2,3-BD in a short period before the complete consumption of these sugars and then appeared to convert 2,3-BD to acetoin. The optimum temperature of the 2,3-BD fermentation was 42 ˚C to 45 ˚C, and the maximum yield of 2,3-BD was 0.3 g/g at 12 h in 20 g/l xylose medium and 0.4 g/g at 6 h in 20 g/l glucose medium at 42 ˚C. The 2,3-BD productivity of the strain was higher than the 2,3-BD productivities of other non-genetically engineered microorganisms reported previously, and the highest productivity was 0.6 g/l·h and 1.2 g/l·h for xylose and glucose, respectively.

**Conclusions:**

Among thermotolerant microbes isolated in Laos, we discovered a strain, *C. sakazakii* OX-25, that can convert xylose and glucose to 2,3-BD with high efficiency and high productivity at high temperatures, suggesting that *C. sakazakii* OX-25 has the potential for industrial application to produce 2,3-BD as an important platform chemical.

**Supplementary Information:**

The online version contains supplementary material available at 10.1186/s12866-022-02577-z.

## Background

In recent years, attention has been given to the use of lignocellulosic biomass as a renewable source for biotechnology because of reduced petroleum resources, greenhouse gas emission risks and fluctuations in crude oil market prices [1, 2]. Most petrochemically synthesized chemicals can be produced using microbial biocatalysts [3]. Of the various microbial fermentation products, 2,3-butanediol (2,3-BD) is a valuable bulk chemical that is used in various application such as production of chemicals, cosmetics, pesticides, foods and pharmaceuticals [3–8]. 2,3-BD is also a widely used antifreeze agent and a valuable fuel additive with a heating value of 27.2 kJ/g, comparable to those of methanol (22.1 kJ/g), n-butanol (33.1 kJ/g) and ethanol (29.1 kJ/g). Due to its high octane number, BD now functions as a gasoline octane booster [7–10]. Furthermore, 2,3-BD can be converted by dehydration to methyl-ethyl-ketone (MEK), a potent fuel additive with higher combustion values and industrial solvents, or to 1,3-butadiene, an important monomer in synthetic rubber production. On the other hand, acetoin, the precursor of or product from 2,3-BD, can be oxidized to diacetyl, which is used as a high-value flavoring of food that gives food a buttery taste and as a bacteriostatic additive for food processing [3, 9]. In the global market, 2,3-BD is expected to grow at a compound annual growth rate of 3% from 2019 to 2027, reaching US $ 220 million by 2027, and the current market price of 2,3-BD is higher than that of 1,4-butanediol [6, 11, 12]. The downstream products of BD have the potential of a global market of around 32 million tons per annually, worth US $ 43 billion [7, 8]. Therefore, the production of bio-based organic chemicals such as BD represents the potential for renewable, sustainable energy for the planet.

In most studies on microbial production of 2,3-BD, food-based materials, including glucose and sucrose, have been used as feedstocks [13, 14], which affect food security. Therefore, 2,3-BD production using lignocellulosic biomass is promising because of its abundant availability, no direct competition with the food supply, and environmental benefits [15, 16]. Since industrial production and use of 2,3-BD are limited by the high cost of its petro-based production, many 2,3-BD-producing microorganisms including bacteria and yeast have been isolated and metabolically engineered to improve 2,3-BD productivity [5, 17, 18]. Most microorganisms prefer glucose as a carbon source when a mixture of different sugars such as sucrose, glucose and fructose is applied [6, 19]. However, there have been a few studies on 2,3-BD production from xylose and responsible microorganisms. Moreover, although high-temperature fermentation is known to have several advantages for reduction of running cost in fermentation [20, 21], most 2,3-BD production has been performed at 30 ˚C and 37 ˚C with xylose and glucose as carbon sources, respectively. In addition to negative effects of high temperature, inhibition of production generally occurs at high substrate concentrations [17].

In this study, we found a strain that quickly utilized xylose but produced almost no xylitol in a screening process for efficient ethanol-producing microorganisms in a xylose medium. 16S rDNA sequencing revealed that the strain was *Cronobacter sakazakii*. The genus *Cronobacter* is generally positive for acetoin production (Voges-Proskauer test) and negative for the methyl red test indicating 2,3-BD rather than mixed acid fermentation [22], but no further study on 2,3-BD production in *C. sakazakii* has been reported. The strain found in this study showed highly efficient production of 2,3-BD using xylose as well as glucose as a carbon source at high temperatures, indicating potential industrial applications.

## Results

### Isolation of xylose-utilizing microbes

About 300 samples that were collected in six provinces of Lao PDR were subjected to enrichment culture at 37 ˚C in a YP2X medium, and the growth of isolated colonies was examined at 37 ˚C, 40 ˚C, 42 ˚C and 45 ˚C on YP2X or YP2D plates. Thirty isolates that were able to grow on YP2X plates at temperatures higher than 40 ˚C were further examined for xylitol accumulation in a test tube containing 2 mL of YP2X liquid medium at 37 ˚C under shaking condition at 100 rpm (Fig. S1). Among almost no xylitol-accumulating strains, one strain was found to exhibit a unique profile of HPLC, and further analysis using authentic compounds as controls revealed that the large peaks at retention times of 22.94 min and 22.23 min of the sample collected at 48 h were 2,3-BD and acetoin, respectively (Fig. S2). 16S rDNA analysis revealed that the sequence of the strain is more than 99% identical to that of *Cronobacter sakazakii* in the GenBank database. Therefore, the strain, named *C. sakazakii* OX-25, was further characterized as follows.

### 2,3-BD and acetoin production in glucose and xylose medium at high temperatures

**Fig. 1:**
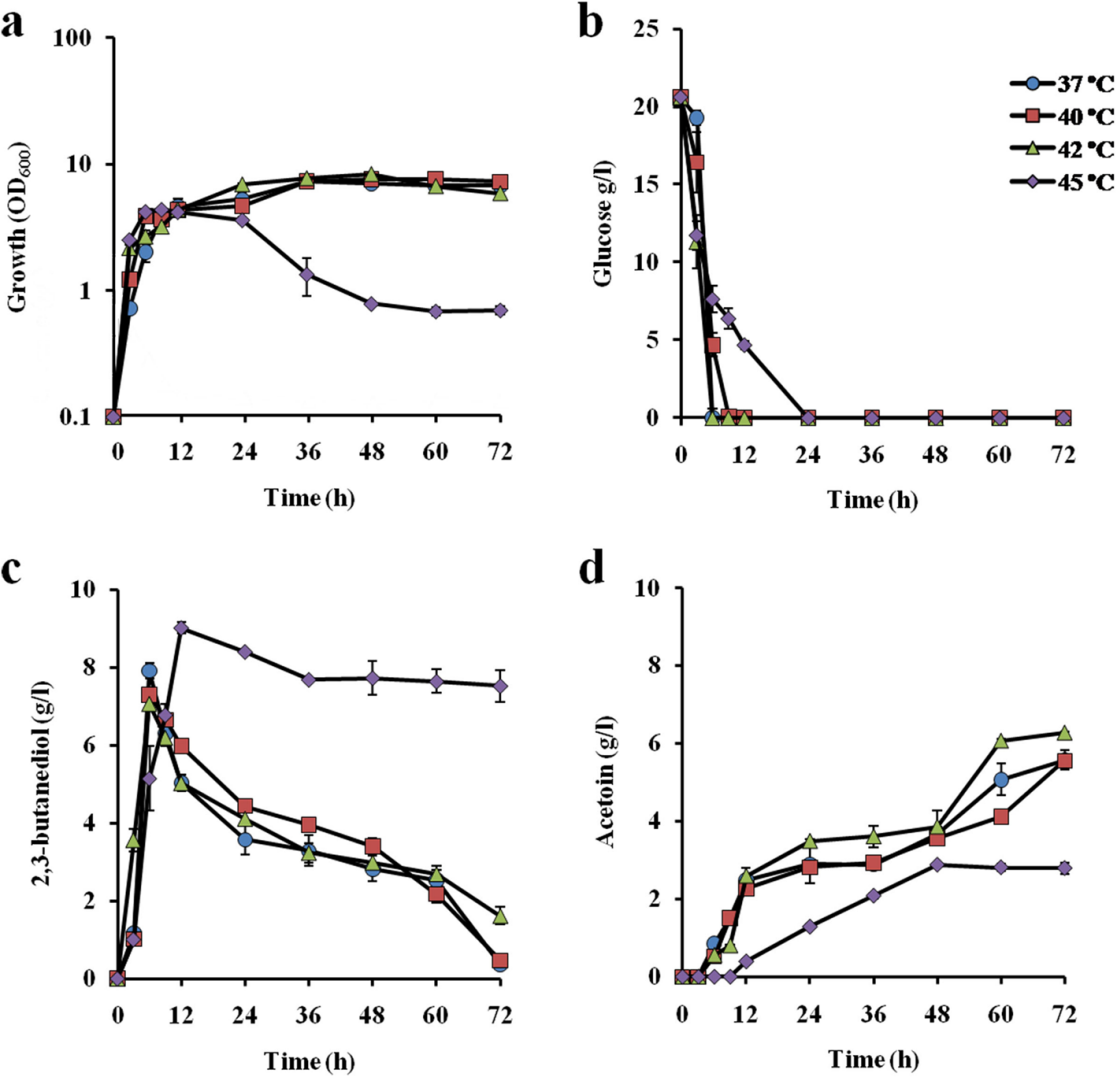
2,3-BD production by *C. sakazakii* OX-25 at different temperatures in a glucose medium. Cells were cultivated in YP medium supplemented with 20 g/l glucose at 37 ˚C (*filled circles*), 40 ˚C (*filled squares*), 42 ˚C (*filled triangles*) and 45 ˚C (*filled diamonds*) under a shaking condition at 100 rpm. **a** Turbidity (OD_600_) and the concentrations of **b** glucose, **c** 2,3-BD and **d** acetoin in the culture medium were determined as described in Materials and methods. Error bars indicate standard deviation of three independent experiments

**Fig. 2:**
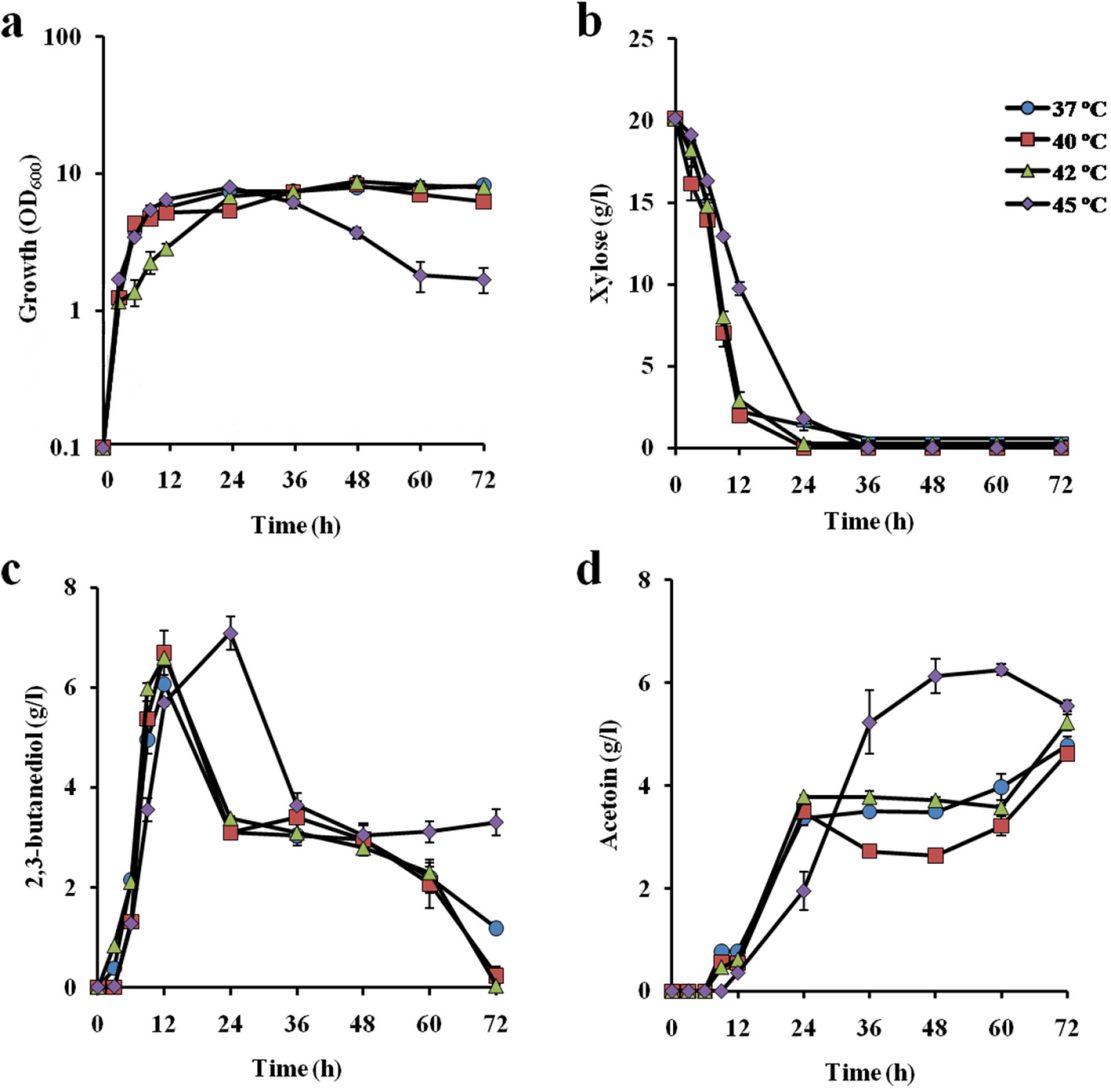
2,3-BD production by *C. sakazakii* OX-25 at different temperatures in a xylose medium. Cells were cultivated in YP medium supplemented with 20 g/l xylose at 37 ˚C (*filled circles*), 40 ˚C (*filled squares*), 42 ˚C (*filled triangles*) and 45˚C (*filled diamonds*) under a shaking condition at 100 rpm. **a** Turbidity (OD_600_) and the concentrations of **b** glucose, **c** 2,3-BD and **d** acetoin in the culture medium were determined as described in Materials and methods. Error bars indicate standard deviation of three independent experiments

2,3-BD and acetoin production abilities in YP medium supplemented with 20 g/l glucose and 20 g/l xylose were examined at different temperatures under a shaking condition (Figs. 1 and 2). In the case of 20 g/l glucose at temperatures of 37 ˚C, 40 ˚C and 42 ˚C (Fig. 1), 2,3-BD increased along with a decrease of glucose, and maximum concentrations of 2,3-BD at the three temperatures were found at 6 h, when glucose was almost completely consumed. The concentrations of 2,3-BD were 7.9 g/l, 7.3 g/l and 7.1 g/l and the productivities were 1.3 g/l·h, 1.2 g/l·h and 1.2 g/l·h at temperatures of 37 ˚C, 40 ˚C and 42 ˚C, respectively. Maximum concentrations of acetoin at the three temperatures were found at 72 h. The increase in acetoin was biphasic, 3-24 h and 36-72 h at 37 ˚C and 40 ˚C and 3-24 h and 48-72 h at 42 ˚C. Interestingly, the decrease in 2,3-BD was biphasic, apparently complementary to the biphasic increase in acetoin. It is speculated that 2,3-BD produced may be a mixture of two forms among (2R, 3R), (2S, 3S) and (2R, 3S) configurations, and two different enzymes that oxidize the individual form are expressed at different conditions during cultivation. At 45 ˚C, the turbidity was reduced after 24 h, indicating cells lysis probably due to the negative effect of the high temperature on cell, while the concentration of 2,3-BD remained high compared to the concentrations at other temperatures tested.

In the case of 20 g/l xylose (Fig. 2), the consumption of xylose was slow compared to that of glucose and the maximum concentrations of 2,3-BD at 37 ˚C, 40 ˚C and 42 ˚C were found at 12 h, when xylose was almost completely consumed. The concentrations of 2,3-BD were 6.1 g/l, 6.7 g/l and 6.6 g/l and the productivities were 0.5 g/l·h, 0.6 g/l·h and 0.6 g/l·h, at 37 ˚C, 40 ˚C and 42 ˚C, respectively. Maximum concentrations of acetoin at the three temperatures were found at 72 h. Decrease in 2,3-BD and increase in acetoin seemed to be biphasic as in the case of glucose as a carbon source. Reduction in turbidity after 24 h was observed as in the case of glucose. The peak of the concentration of 2,3-BD at 45 ˚C was delayed compared to that at other temperatures tested and the accumulation of acetoin was monophasic with a peak at 60 h.

### 2,3-BD and acetoin production with high concentrations of glucose or xylose

**Fig. 3:**
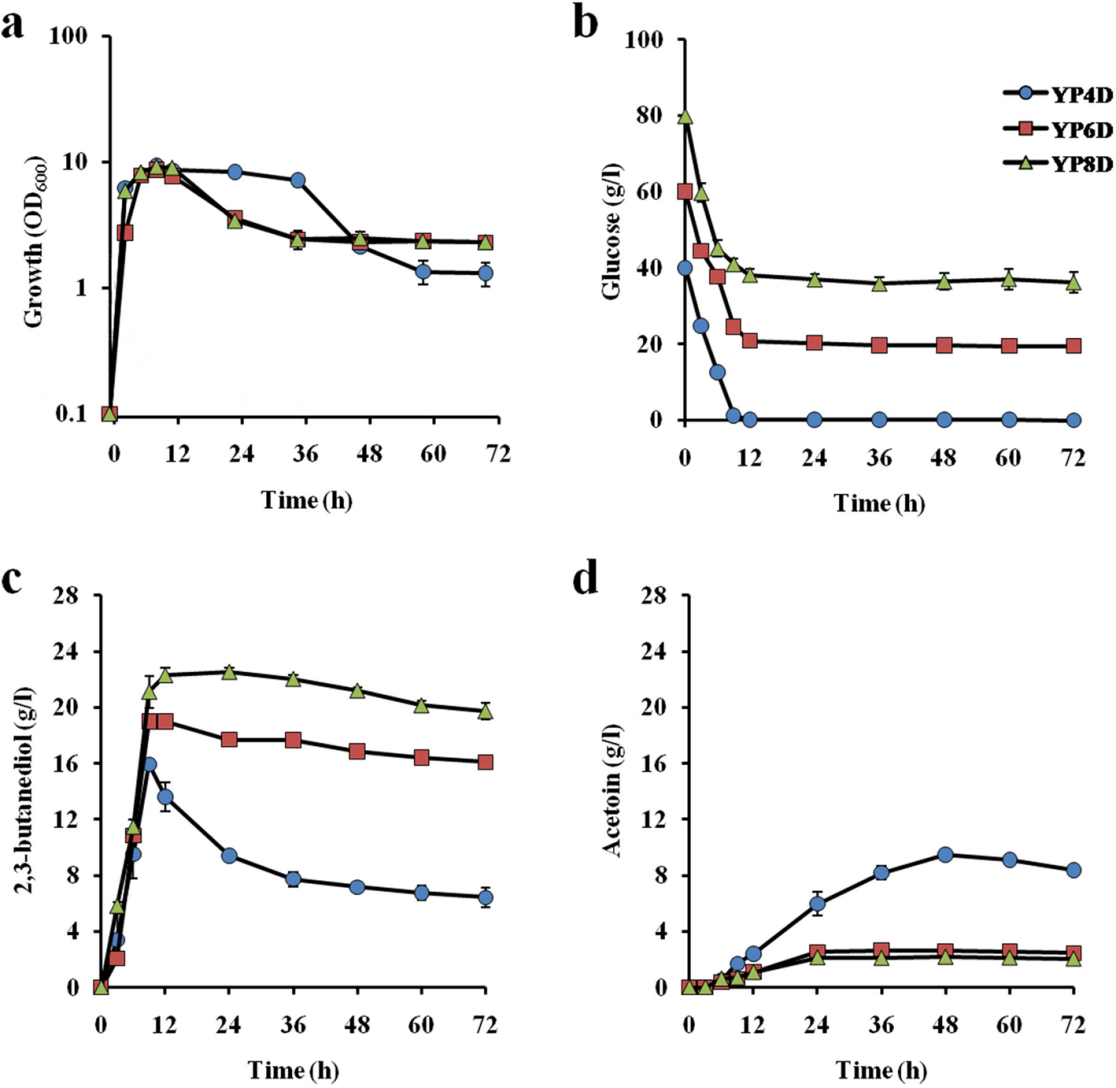
Effects of initial glucose concentrations on 2,3-BD production. Cells were cultivated in YP medium supplemented with 40 g/l (*filled circles*), 60 g/l (*filled squares*) or 80 g/l (*filled triangles*) glucose at 42˚C under a shaking condition at 100 rpm. **a** Turbidity (OD_600_) and the concentrations of **b** glucose, **c** 2,3-BD and **d** acetoin in the culture medium were determined as described in Materials and methods. Error bars indicate standard deviation of three independent experiments

To further understand the production capacity of 2,3-BD and acetoin, higher concentrations of glucose or xylose were examined at 42 ˚C because the productivity of 2,3-BD was nearly the same as the productivities at 37 ˚C and 40 ˚C (Figs. 1 and 2). When 40 g/l, 60 g/l and 80 g/l glucose were applied, 16 g/l and 19 g/l of 2,3-BD at 9 h and 22 g/l of 2,3-BD at 12 h were produced, respectively, and 9 g/l, 3 g/l and 2 g/l of acetoin were produced at 48 h, respectively (Fig. 3). However, 20 g/l and 36 g/l glucose remained in the medium under the condition with 60 g/l and 80 g/l glucose. The largest amounts of 2,3-BD were detected at 9 h with 80% and 63% of the theoretical yield and at 12 h with 56% of the theoretical yield under the conditions with 40 g/l, 60 g/l and 80 g/l glucose, respectively. The sum of 2,3-BD and acetoin at 12 h under the 80 g/l glucose condition was 59% of the theoretical yield, which was much lower than 88% and 66% at 9 h under the 40 g/l and 60 g/l glucose conditions, and the conversion of 2,3-BD to acetoin seemed to be stopped after 24 h, suggesting that the production of 2,3-BD and the conversion of 2,3-BD to acetoin were prevented. These findings suggest that about 22 g/l of 2,3-BD is the upper limit in the case of glucose as a carbon source. Notably, in contrast to the experiments with 20 g/l glucose, both the decrease in 2,3-BD and increase in acetoin were monophasic, and the 2,3-BD decrease was not significant in experiments with 60 g/l and 80 g/l glucose. It is presumed that the conversion of 2,3-BD to acetoin was prevented when glucose remained in the medium.

**Fig. 4:**
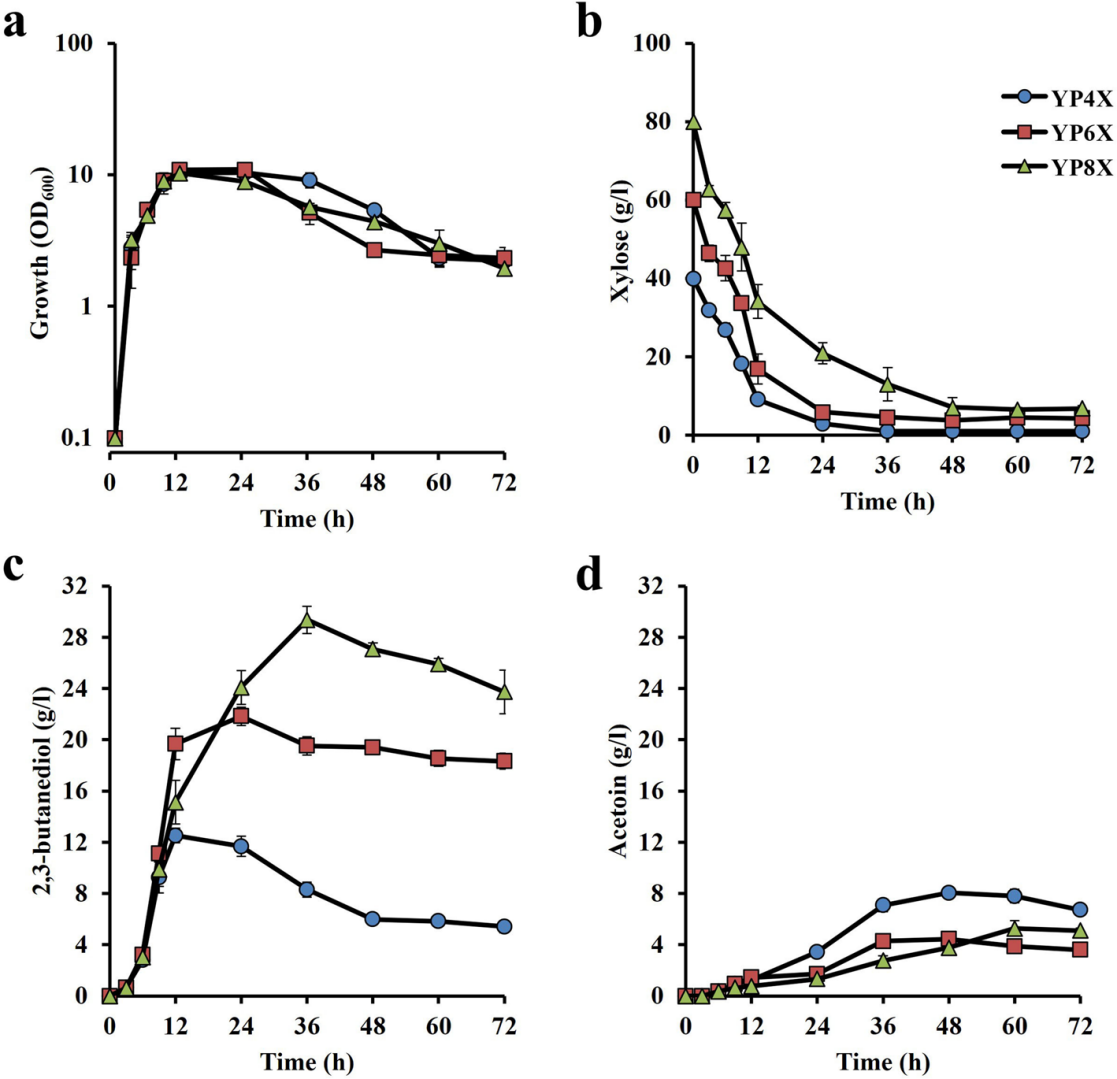
Effects of initial xylose concentrations on 2,3-BD production. Cells were cultivated in YP medium supplemented with 40 g/l (*filled circles*), 60 g/l (*filled squares*) or 80 g/l (*filled triangles*) xylose at 42˚C under a shaking condition at 100 rpm. **a** Turbidity (OD_600_) and the concentrations of **b** glucose, **c** 2,3-BD and **d** acetoin in the culture medium were determined as described in Materials and methods. Error bars indicate standard deviation of three independent experiments

When 40 g/l, 60 g/l and 80 g/l xylose were applied, 13 g/l of 2,3-BD at 12 h, 22 g/l at 24 h and 29 g/l at 36 h were produced, respectively, and 8 g/l of acetoin at 48 h, 4 g/l at 48 h and 5 g/l at 60 h were produced, respectively (Fig. 4). However, 4 g/l xylose and 7 g/l xylose remained in the medium at 48 h under the condition with 60 g/l and 80 g/l xylose, respectively. The largest amounts of 2,3-BD were found at 12 h with 53% of the theoretical yield, at 24 h with 60% of the theoretical yield and at 36 h with 59% of the theoretical yield under the conditions with 40 g/l, 60 g/l and 80 g/l xylose, respectively. The sums of 2,3-BD and acetoin under the 60 g/l and 80 g/l xylose conditions were 65% (at 24 h) and 65% (36 h) of the theoretical yield, respectively, which were much higher than 58% at 12 h under the 40 g/l xylose condition, but the conversion of 2,3-BD to acetoin at high xylose concentrations was greatly reduced. These findings suggest that this strain is able to produce about 29 g/l of 2,3-BD in the case of xylose as a carbon source. Therefore, it is assumed that the production of 2,3-BD with xylose is greater than that with glucose, although the production speed of the former is slower than that of the latter.

### 2,3-BD and acetoin production with mixed sugars of glucose and xylose

Considering cellulosic biomass, the production of 2,3-BD and acetoin from mixed sugars of glucose and xylose was examined using three different combinations of both sugars (Fig. S3). Surprisingly, xylose was hardly utilized even after glucose had been completely consumed. When 20 g/l, 40 g/l and 60 g/l glucose in addition to 20 g/l xylose were applied, 8 g/l of 2,3-BD at 6 h, 17 g/l at 12 h and 22 g/l at 12 h were produced, respectively, and 4 g/l of acetoin at 24 h, 3 g/l at 24 h and 2 g/l at 24 h were produced, respectively. One of the reasons for xylose utilization inability in the presence of glucose may be glucose repression. Considering the findings that the strain was found to be potent in using xylose than glucose in fermentation using individual sugars, it is speculated that enzymes related to xylose uptake and catabolism are negatively regulated in the presence of glucose, resulting in prevention of xylose utilization. Further research is required for improvement of the inability.

## Discussion

During the process to isolate efficiently ethanol-fermenting thermotolerant microbes on xylose, we discovered a strain, *C. sakazakii* OX-25, that produced mainly 2,3-BD at the early growth phase. *C. sakazakii* OX-25 was then found to have several superior characteristics to other 2,3-BD-fermenting microbes. Compared to previously reported data for other wild-type microbes (Table 1), *C. sakazakii* OX-25 can utilize both glucose and xylose and showed the highest productivity when glucose or xylose was used as a carbon source. *Klebsiella pneumoniae* PM2 and *Paenibacillus polymyza* DSM 365 can also utilize both sugars, but their productivities were much lower than those of *C. sakazakii* OX-25 [23, 24]. *C. sakazakii* OX-25 was capable of efficiently fermenting 2,3-BD. Its production levels of 2,3-BD from glucose or xylose were higher than or equivalent to those of other microbes except for *P. polymyza* DSM 365. Moreover, this strain was able to produce the compound from both sugars at high temperatures. Fermentation experiments with other microbes were carried out at temperatures of 30 ˚C to 37 ˚C, except for *Bacillus licheniformis* YNP5-TSU [25]. Recently, the production of 2,3-BD by *P. polymyxa* DSM 742 has been reported, and the titer of 2,3-BD was 10.57 g/l (0.26 g/g) at 30 ˚C for 40 h in a medium containing 30 g/l glucose [26]. Furthermore, the metabolically engineered *E. ludwigii* has been shown to achieve 7.1 g/l (0.35 g/g) of 2,3-BD at 30 ˚C for 20 h in a medium containing 20 g/l of glucose [7]. Meanwhile, *C. sakazakii* OX-25 achieved 7.1 g/l (0.4 g/g) at 42 ˚C for 6 h in a medium containing 20 g/l glucose. These results indicate that newly isolated *C. sakazakii* OX-25 has a high potential for 2,3-BD production.

**Table 1:** Comparison of the amount of 2,3-BD produced by *C. sakazakii* OX-25 and the amounts produced by various microbes

Strain	Conc. (g/l)	Temp. (ºC)	Time (h)	2,3-BD (g/l)	2,3-BD (g/g)	2,3-BD (g/l·h)	Reference
*Cronobacter sakazakii* OX-25	Xyl: 20	42	12	6.6	0.3	0.6	This study
	Xyl: 40	42	12	12.5	0.3	1	This study
	Xly: 60	42	24	21.8	0.4	0.9	This study
	Xyl: 80	42	36	29.4	0.4	0.8	This study
*Enterobacter ludwigii*	Xyl: 20	30	17	7.6	0.4	0.4	[27]
	Xyl: 40	30	40	~ 12	0.3	0.3	[27]
	Xly: 60	30	40	~ 10	0.2	0.3	[27]
	Xyl: 80	30	40	~ 8	0.1	0.2	[27]
*Klebsiella pneumoniae* PM2	Xyl: 20	30	36	6	0.3	0.2	[23]
	Xyl: 40	30	36	13	0.3	0.4	[23]
	Xyl: 60	30	36	20	0.3	0.6	[23]
	Xyl: 80	30	36	25	0.3	0.7	[23]
*Paenibacillus polymyxa* DSM 365	Xyl: 55.9	35	70	29.4	0.3	0.4	[24]
*Cronobacter sakazakii* OX-25	Glu: 20	42	6	7.1	0.4	1.2	This study
	Glu: 40	42	9	15.9	0.4	1.8	This study
	Glu: 60	42	9	19	0.3	2.1	This study
	Glu: 80	42	9	21.1	0.3	2.3	This study
*Klebsiella oxytoca* M1	Glu: 42.3	30	48	10.4	0.2	0.2	[28]
*Bacillus licheniformis* DSM 8785	Glu: 20	30	15	1.6	0.3	0.4	[29]
	Glu: 40	30	20	2.0	0.4	0.7	[29]
	Glu: 60	30	25	1.2	0.4	0.9	[29]
	Glu: 80	30	31.5	1.5	0.4	1.0	[29]
*Klebsiella pneumoniae* PM2	Glu: 20	30	24	8.0	~ 0.4	~ 0.3	[23]
	Glu: 40	30	24	18.0	0.5	0.8	[23]
	Glu: 60	30	36	21.8	0.4	0.6	[23]
	Glu: 80	30	36	26.8	0.3	0.7	[23]
*Paenibacillus polymyxa* DSM 365	Glu: 38.5	35	60	32.3	0.3	0.5	[24]
*Acillus amyloliquefaciens*	Glu: 40	37	20	4.5	0.1	0.2	[24]
	Glu: 60	37	26	11.5	0.2	0.4	[24]
	Glu: 80	37	36	17.7	0.2	0.5	[24]
*Enterobacter cloacae* SDM	Glu: 40	37	10	~ 18	0.5	1.8	[30]
*Bacillus licheniformis* YNP5-TSU	Glu: 48.3	50	18	20.5	0.4	0.29	[25]

Fermentation profiles revealed that *C. sakazakii* OX-25 accumulated 2,3-BD at the early growth phase and accumulated acetoin at a relatively late growth phase (Figs. 1 and 2). Since both of the compounds are located next to each other in the metabolic pathway, 2,3-BD may be oxidized to acetoin. The conversion of 2,3-BD to acetoin was initiated when glucose or xylose as the carbon source had been almost completely consumed, suggesting that accumulated 2,3-BD was taken up into cells. A part of the resultant acetoin may be further oxidized and the majority of acetoin was exported outside the cells. Notably, during cultivation, there were two-time reductions of 2,3-BD (for example, at 6-24 h and 36-72 h in the 20 g/l glucose medium at 37 ˚C) and at 12-24 h and 48-72 h in the 20 g/l xylose medium at 37 ˚C and two-time increases of acetoin (for example, at 3-24 h and 36-72 h in the case of the 20 g/l glucose medium at 37 ˚C, and at 6-24 h and 48–72 h in the 20 g/l xylose medium at 37 ˚C) (Figs. 1 and 2). It is thus thought that accumulated 2,3-BD may be a mixture of at least two isomers as in most other microbes [9, 29, 31] and that one of them is first oxidized to acetoin and the other is oxidized later.

When glucose was used as a carbon source, 2,3-BD production by the strain reached 80% of the theoretical yield at 9 h in the condition with 40 g/l glucose, but the amounts of 2,3-BD produced were reduced to 63% and 56% of the theoretical yield at 9 and 12 h in the conditions with 60 g/l and 80 g/l glucose, respectively (Fig. 3). On the other hand, in the case of xylose as a carbon source, the 2,3-BD production by the strain reached 59% of the theoretical yield at 36 h in the condition with 80 g/l xylose, but the amount of 2,3-BD produced was reduced to 53% of the theoretical yield at 12 h in the condition with 40 g/l xylose (Fig. 4). Notably, cell turbidity declined after 12 h and 24 h in the glucose medium and xylose medium, respectively, at 45 ˚C (Figs. 1 and 2) and after 12 h or 36 h in the media containing high sugar concentrations even at 42 ˚C (Figs. 3 and 4), suggesting a negative impact of high temperatures or substrate inhibition. The latter is consistent with previous reports [17].

Compared to the previously reported 2,3-BD fermentation by other microbes, *C. sakazakii* OX-25 was found to have several useful features including high productivity and good performance at high temperatures. Additionally, it showed almost the same yield as that of other efficient 2,3-BD-producing microbes. These features indicate that this strain is suitable and promising for the production of 2,3-BD. Considering the further conversion of 2,3-BD to acetoin, which may be due to consumption of sugars such as glucose or xylose in the medium, the production level of 2,3-BD could be maintained by keeping minimum amounts of sugars. *Klebsiella*, *Enterobacter*, and *Serratia* genera [17] also produce about the same amount of 2,3-BD as that produced by *C. sakazakii* OX-25. However, their pathogenicity (genera of risk group 2) has been pointed out as an obstacle for industrial use. In this regard, *C. sakazakii* is less pathogenic and is classified as risk group 1 according to the guidelines of the US National Institutes of Health. Furthermore, *C. sakazakii* OX-25 was able to efficiently produce 2,3-BD at high temperatures, as opposed to most 2,3-BD fermentations with other microbes at 30 ˚C. Compared to low-temperature fermentation around 30 ˚C, high-temperature fermentation around 40 ˚C has several advantages such as reduction of operating costs for cooling systems, minimization of the risk of contamination, and efficient achievement of simultaneous saccharification and fermentation [32–34]. Taken together, the characteristics of *C. sakazakii* OX-25 enable energy-saving and cost-effective production of 2,3-BD. In particular, its high productivity and high temperature fermentation with the strain may be new important factors in 2,3-BD production in terms of process economics.

## Conclusions

In the production of 2,3-BD, microbes that can utilize cellulosic biomass and ferment quickly and efficiently are extremely important for industrial applications. In this study, we succeeded in isolating a xylose-fermenting microbe, *C. sakazakii* OX-25, which has such potential. The microbe was able to utilize xylose and glucose as a carbon source and produced 2,3-BD at relatively high speed at high temperatures. When 20 g/l and 40 g/l glucose were applied, 7.1 g/l (0.4 g/g or 1.2 g/l·h) of 2,3-BD at 42 ˚C for 6 h and 15.9 g/l (0.4 g/g or 1.8 g/l·h) of 2,3-BD at 42 ˚C for 9 h, respectively, were achieved. In the cases of 20 g/l and 40 g/l xylose, 6.6 g/l (0.3 g/g or 0.6 g/l·h) of 2,3-BD at 42 ˚C for 12 h and 12.5 g/l (0.3 g/g or 1 g/l·h) of 2,3-BD at 42 ˚C for 12 h, respectively, were achieved. The theoretical yields reached 71%, 80%, 55% and 53% under the conditions with 20 g/l glucose, 40 g/l glucose, 20 g/l xylose and 40 g/l xylose, respectively. These results suggest that *C. sakazakii* OX-25 shows the highest productivity of previously reported non-genetically engineered microorganisms, suggesting that the strain has the potential for 2,3-BD production in industrial applications.

## Materials and methods

### Strain, media and growth conditions

*C. sakazakii* OX-25 was isolated by enrichment culture at 37 ˚C from rotten grass in Laos and its isolation with other strains will be reported elsewhere. Cells were grown in YP medium (10 g/l yeast extract (Difco) and 20 g/l peptone (Difco) containing 20 g/l glucose (YP2D) or 20 g/l xylose (YP2X) at 37 ˚C, 40 ˚C or 42 ˚C under a shaking condition at 160 rpm. The thermotolerance was examined by checking the growth at 37 ˚C, 40 ˚C, 42 ˚C and 45 ˚C on YPD or YPX plates as described previously [35].

### Isolation of xylose-utilizing microorganisms

Xylose-utilizing microorganisms were isolated from various samples in six provinces in Laos (latitude and longitude is 18° 00’N and 105° 00’ E): Luangphrabang (lat 16° 27’ 21’’ N; lng 108° 38’10’’ E), Oudomxay (lat 20° 30’ 15’’ N; lng 101° 50’ 22’’ E) and Xiengkhuang (lat 19° 37’ 0’’ N; lng 103° 33’ 0’’ E) that are located at the northern part of Laos; Vientiane (lat 18° 04’ 05’’ N; lng 102° 40’ 43’’ E) and Bolikhamxay (lat 18° 24’ 2’’ N; lng 104° 14’ 2’’ E) that are located at the central part; Champasak (lat 15° 3’ 32’’ N; lng 106° 39’ 11’’ E) that is located at the southern part of the country. The samples included vegetables, dried banana and papaya leaves, grass, rice straw, sawdust, coconut and other fruit shells, and rice starch waste of noodles. Isolation was carried out at 37 ˚C by enrichment cultures for all samples except for ripened fruits, which were subjected to non-enrichment cultures as described previously [32, 35]. In the enrichment cultures, samples (5 to 10 g) were pressed into small pieces and transferred into 100-ml Erlenmeyer flasks containing 25 ml of YPD medium and then incubated at 37 ˚C for 24-48 h under a shaking condition at 160 rpm. In the non-enrichment cultures, samples (5 to 10 g) of ripened fruits were pressed into small pieces and transferred into small glass bottles. Distilled water was then added, and the bottles were covered with aluminum foil and incubated at 37 ˚C for 24-48 h. Both of the cultures were then streaked on YPX agar plates and incubated at 37 ˚C for 24 h to 48 h, and single colonies were obtained for further experiments.

### Analysis of 2,3-BD fermentation

To examine growth and fermentation parameters, YP medium supplemented with glucose at 20 g/l (YP2D), 40 g/l (YP4D), 60 g/l (YP6D) or 80 g/l (YP8D), YP medium supplemented with xylose at 20 g/l (YP2X), 40 g/l (YP4X), 60 g/l (YP6X) or 80 g/l (YP8X) and YP medium supplemented with 20 g/l glucose + 20 g/l xylose (YP2D2X), 40 g/l glucose + 20 g/l xylose (YP4D2X) or 60 g/l glucose + 20 g/l xylose (YP6D2X) were used. Strains were pre-cultured in YPD medium at 30 ˚C under a shaking condition at 160 rpm for 18 h. The pre-culture was inoculated into a 100-ml flask containing 30 ml of liquid medium at an optical density (OD_600_) of 0.1, followed by incubation at temperatures of 37 ˚C, 40 ˚C, 42 ˚C and 45 ˚C. Cell density was determined by measurement on a UV-VIS spectrophotometer (Shimadzu, Japan). Fermentation parameters were analyzed by a high-performance liquid chromatography (HPLC) system (Hitachi, Japan), as described previously [34, 36], consisting of a Hitachi Model D-2000 Elite HPLC system Manager, L-2130 column oven, L-2130 pump, L-2200 auto-sampler and L-2490 RI detector equipped with a GL-C610H-S gel pack column at 60 ˚C with 0.5 mL/min eluent of 0.1% phosphoric acid. Authentic 2,3-BD (Wako, Japan) and acetoin (TCI, Japan) were used as controls.

### Identification of selected strains

Isolation of genomic DNA and amplification and determination of 16S rDNA were performed according to the methods of Green and Sambrook [37]. The region of 16S rDNA was amplified by PCR using Ex Taq polymerase (Takara, Japan) and primers that are generally utilized: rDNA forward, 5’-AGAGTTTGATCCTGGCTCAG-3’ and reverse, 5’-GGTTACCTTGTTACGACTT-3’, generating a 0.8-kb band in 1.6% agarose gel [38]. The band was extracted by using a QIAquick gel extraction kit (QIAGEN) and subjected to DNA sequencing by the Sanger method [39].

## Supplementary Information

**Additional file 1.**

## Data Availability

The sequence of the part of 16S rDNA in *Cronobacter sakazakii* OX-25 has been deposited to the GenBank (NCBI) under the accessory number ON306760. Other data generated or analyzed during this study are included in this published article and its supplementary information file.
