# Supplementary material for: Highly efficient production of 2,3-butanediol from xylose and glucose by newly isolated thermotolerant Cronobacter sakazakii
Source: BMC Microbiol. 2022 Jun 24;22:164. doi: 10.1186/s12866-022-02577-z (PMC9229127; doi:10.1186/s12866-022-02577-z)
Supplement: Supplementary file 1 — Additional file 1. [file 12866_2022_2577_MOESM1_ESM.docx]

**Supplementary Materials**

**Highly efficient production of 2,3-butanediol from xylose and glucose by newly isolated thermotolerant *Cronobacter sakazakii***

Chansom Keo-oudone^1†^, Koudkeo Phommachan^2†^, Orathai Suliya^1^, Mochamad Nurcholis^3^, Somchanh Bounphanmy^1^, Tomoyuki Kosaka^2,45^ and Mamoru Yamada^2,4,5*^

*^1^ Department of Biology, Faculty of Natural Science, National University of Laos, Lao PDR. Vientiane 7322, Laos.*

*^2^ Graduate School of Sciences and Technology for Innovation, Yamaguchi University, Yamaguchi 753-8515, Japan.*

*^3^ Department of Food Science and Technology, Faculty of Agricultural Technology, Brawijaya University, Malang 65145, Indonesia.*

*^4^ Department of Biological Chemistry, Faculty of Agriculture, Yamaguchi University, Yamaguchi 753-8515, Japan.*

*^5^ Research Center for Thermotolerant Microbial Resources, Yamaguchi University, Yamaguchi 753-8515, Japan.*

^†^These authors contributed equally.

* Corresponding to Mamoru Yamada: [m-yamada@yamaguchi-u.ac.jp](mailto:m-yamada@yamaguchi-u.ac.jp)


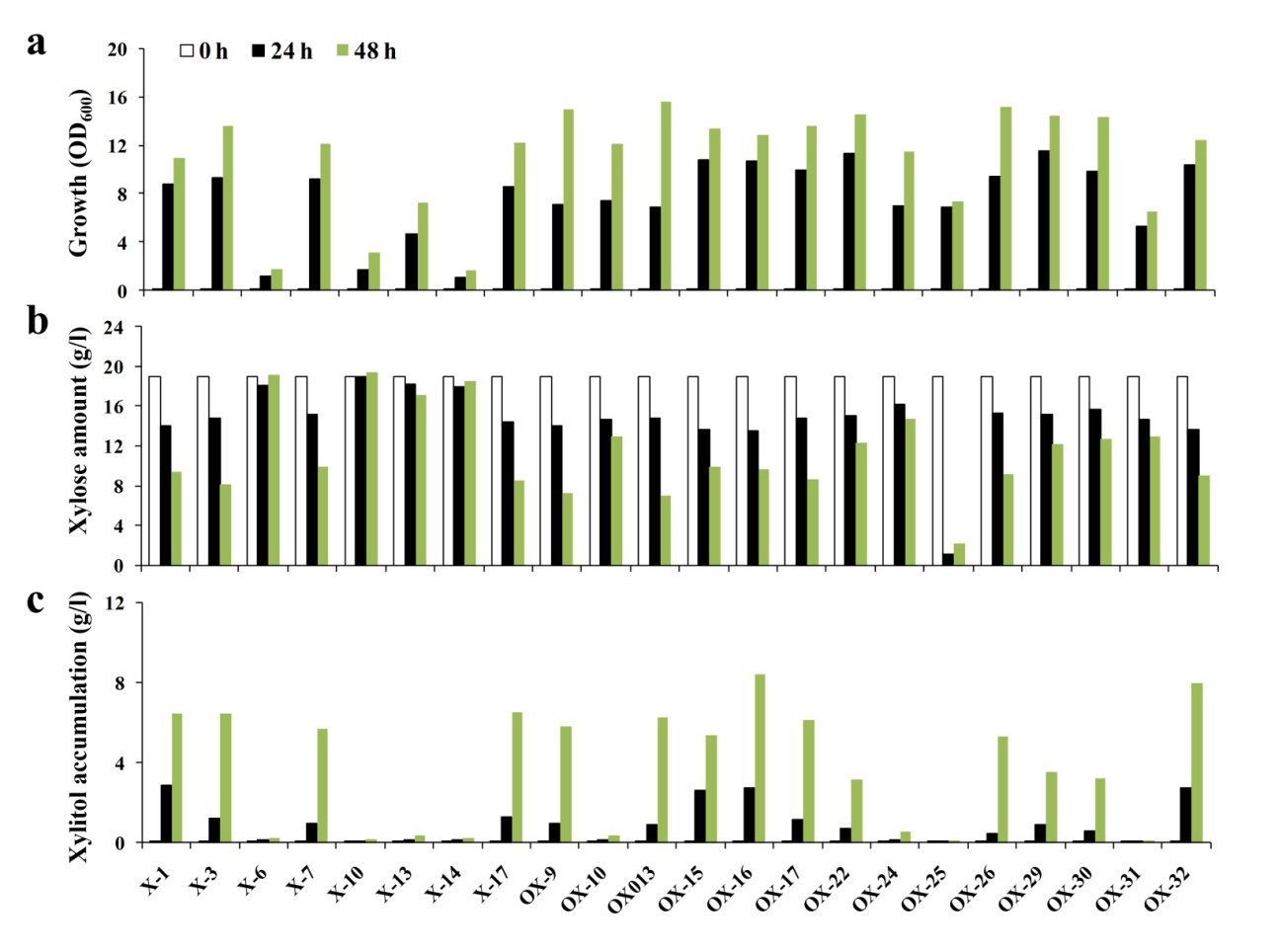
**Figure S1.** Screening strains with abilities for high xylose consumption and low xylitol accumulation. The screening was performed by cultivation in test tubes that each contained 3 mL of YP medium supplemented with 20 g/l xylose at 37 ˚C under a shaking condition at 100 rpm. **a** Turbidity (OD_600_) and the concentrations of **b** xylose and **c** xylitol in the culture medium were determined as described in Materials and methods.

**
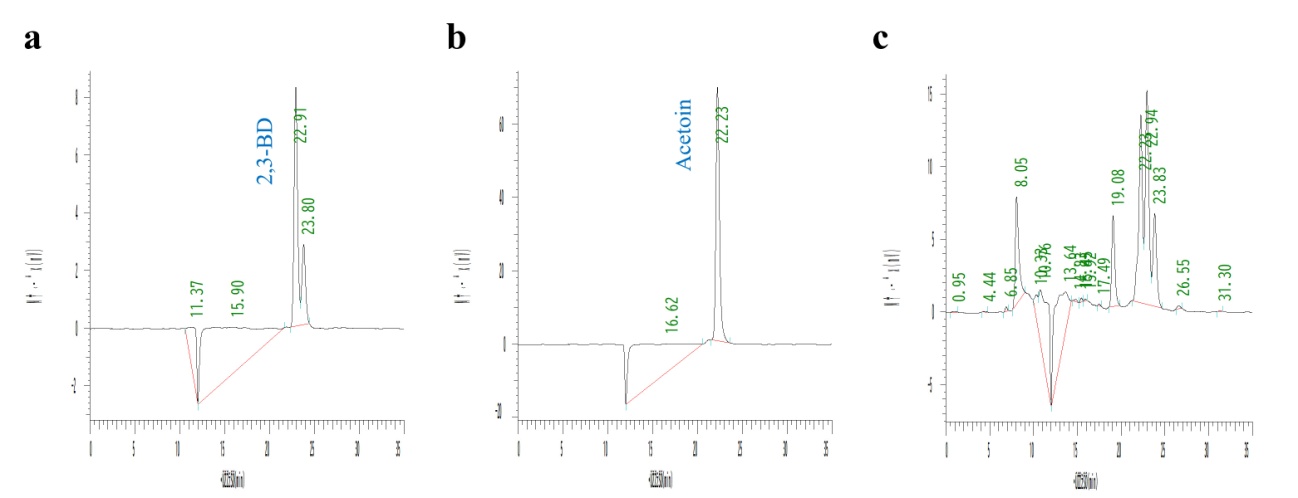
**

**Figure S2.** HPLC profiles showing the peaks of authentic 2,3-BD and acetoin and compounds in one sample. **a** HPLC profile showing a peak of authentic 2,3-BD, **b** HPLC profile showing a peak of authentic acetoin and **c** HPLC profile of one sample taken at 48 h showing the peaks of 2,3-BD and acetoin at retention times of 22.94 min and 22.23 min, respectively.

**
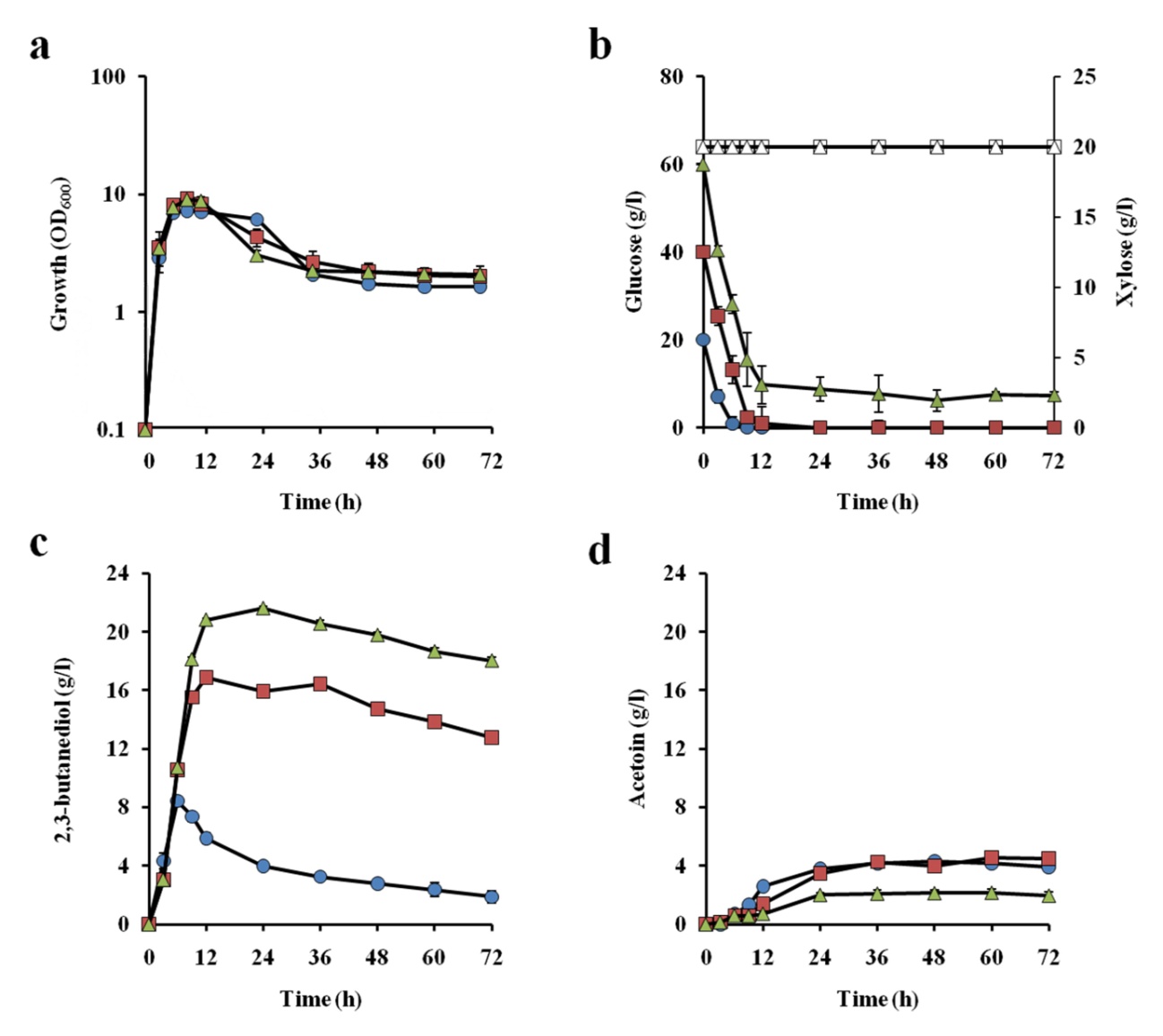
**

**Figure S3.** 2,3-BD production by *C. sakazakii* OX-25 on mixed sugars at 42˚C. Cells were cultivated at 42˚C in YP medium supplemented with glucose and xylose at ratios of 1:1, 2:1 or 3:1 under a shaking condition at 100 rpm. The concentration of xylose was fixed at 20 g/l and the concentrations of glucose were 20 g/l (*filled circles* or *opened circles*), 40 g/l (*filled squares* or *opened squares*) and 60 g/l (*filled triangles* or *opened triangles*). **a** Turbidity (OD_600_) and the concentrations of **b** glucose and xylose, **c** 2,3-BD and **d** acetoin in the culture medium were determined as described in Materials and methods. Closed symbols represent glucose consumption and opened symbols represent xylose consumption. Error bars indicate standard deviation of three independent experiments.
